# Cell-free DNA measurement of three genomes after allogeneic MSC therapy in kidney transplant recipients indicates early cell death of infused MSC

**DOI:** 10.3389/fimmu.2023.1240347

**Published:** 2023-11-02

**Authors:** Geertje J. Dreyer, Jos JM. Drabbels, Johan W. de Fijter, Cees van Kooten, Marlies EJ. Reinders, Sebastiaan Heidt

**Affiliations:** ^1^ Department of Internal Medicine (Nephrology) and Transplant Center, Leiden University Medical Center, Leiden, Netherlands; ^2^ Department of Immunology, Leiden University Medical Center, Leiden, Netherlands; ^3^ Department of Internal Medicine, Nephrology and Transplantation, Erasmus MC Transplant Institute, Erasmus University Medical Center, Rotterdam, Netherlands

**Keywords:** kidney transplansplantation, mesenchymal stromal cell (MSC), cell free DNA (cfDNA), allogeneic MSC, third party MSC

## Abstract

**Introduction:**

Mesenchymal stromal cell (MSC) therapy is a promising treatment that allows for drug minimization in clinical kidney transplantation. While it is thought that MSCs rapidly go into apoptosis after infusion, clinical evidence for this is scarce since methods to detect cell death of infused cells in vivo are lacking. Cell-free DNA (cfDNA) has recently gained attention as a biomarker for cell death.

**Methods:**

In this study, we longitudinally measured cfDNA in plasma samples of the recipient, kidney donor, and allogeneic third-party MSC in the context of the Neptune study. cfDNA levels were measured at several time points before and after allogeneic MSC infusion in the 10 recipients who participated in the Neptune study. cfDNA ratios between the recipient, kidney graft, and MSC were determined.

**Results:**

We observed a peak in MSC-derived cfDNA 4 h after the first and second infusions, after which MSC-derived cfDNA became undetectable. Generally, kidney graft-derived cfDNA remained in the baseline-level range.

**Discussion:**

Our results support preclinical data that MSC are short-lived after infusion, also in a clinical in vivo setting, and are relevant for further research into the mechanism of action of MSC therapy.

MEJ Reinders was affiliated with Leiden University Medical Center at the time of the trial and is currently affiliated with Erasmus University Medical Center.

## Introduction

Mesenchymal stromal cell (MSC) therapy has gained significant interest in kidney transplantation in recent years. MSC therapy has been investigated in several clinical research settings, either as induction therapy, treatment of acute rejection, or support of maintenance therapy allowing weaning of immunosuppressive drugs (1–5). In the setting of kidney transplantation, for most clinical studies, autologous MSC therapy has been applied (3, 5–7). However, since it takes several weeks to manufacture an MSC product, the use of “off-the-shelf” allogeneic MSC would be more feasible in the clinical setting. In the Neptune study, allogeneic MSCs were infused 6 months after transplantation (8). In this phase 1b study, third-party MSCs were selected to have no repeated human leucocyte antigen (HLA) mismatches with the kidney donor to minimize the risk of an anti-donor immune response. The study proved the safety of infusion of HLA-selected third-party MSC in kidney transplant recipients in combination with lower tacrolimus trough levels after infusion (pre-MSC infusion 6.1 (± 1.7) ng/mL versus post-MSC infusion 3.0 ( ± 0.9) ng/mL).

MSCs are thought to promote immunological tolerance after transplantation and to have immune-modulatory and anti-inflammatory properties (4, 9, 10). However, the mechanism of action of MSC therapy is still not fully elucidated. Preclinical murine studies suggested that a potential local mechanism of action is unlikely since most MSCs accumulate in the microvasculature of the lungs and are undetectable within a few hours after infusion (11, 12). Several studies suggest the secretion of paracrine-acting factors such as cytokines, growth factors, and immunomodulatory proteins (13–16). Another suggested mechanism of action is that MSCs are phagocytosed by monocytes in the lungs and that these monocytes play an important role in the mediation, distribution, and transmission of the immunomodulatory effect of the MSC (17). The murine study of the group of Dazzi et al. confirmed that MSCs are degraded shortly after infusion (10). Additionally, they discovered that the process of apoptosis is crucial for the immunomodulatory effect of MSC. It is assumed that this is in part dependent on phagocyte-derived indoleamine 2,3-dioxygenase (IDO) activity upon engulfment of apoptotic MSC. Despite these preclinical data, proof of cell death of MSC upon infusion in the clinical setting is scarce.

Recently, cell-free DNA (cfDNA) has been identified as an interesting biomarker for rejection in solid organ transplantation (18). The presence of cfDNA is partly due to active secretion, but the most important source is cells undergoing apoptosis or necrosis. Therefore, donor-derived cfDNA can be used as a readout of cell damage and cell death and as an indirect measure for graft rejection (19–21). In 2017, the results of the DART trial were published (22). In this study, donor-derived cell-free DNA (dd-cfDNA) was measured after kidney transplantation and used as a biomarker of biopsy-confirmed acute rejection. The results discriminated best between antibody-medicated rejection (ABMR) and no rejection, with median dd-cfDNA values of 2.9% and 0.3%, respectively. The ADMIRAL study showed that the rise of dd-cfDNA (levels of ≥0.5%) was significantly correlated with both clinical and subclinical rejection. Moreover, this study showed that these levels of dd-cfDNA were associated with an almost threefold increase in the risk of *de novo* donor-specific antibody (DSA) development (23). More recently, the results of the TRIFECTA trial were published (24). This study also concluded that dd-cfDNA (%) was strongly associated not only with active rejection, particularly ABMR, but also the most active T-cell-mediated rejection. Therefore, cfDNA appears to be a sensitive biomarker for allogeneic cell death, although its specificity for the diagnosis of acute rejection is not yet good enough to completely replace the invasive kidney biopsy (25).

In the human setting, the fate and working mechanism of MSC remain unclear. In the current study, we measured patient, kidney donor, and MSC donor-specific cfDNA levels at several time points after MSC infusion as an indirect measure of allogeneic MSC cell death *in vivo*. Since we used third-party MSC, this study is the first to separate cfDNA for three genomes: the recipient, the kidney donor, and the MSC donor. Our results confirm the short life span of MSC in humans upon infusion.

## Materials and methods

### Patients

For the current study, all 10 patients treated with allogeneic MSC in the context of the Neptune study were included (8, 26). Patients were between 24 and 68 years old and recipients of a first kidney graft from a living donor. Patients received alemtuzumab induction therapy with maintenance therapy consisting of prednisone, tacrolimus (Advagraf^®^), and everolimus. Six months after kidney transplantation (weeks 25 and 26), patients received two doses of allogeneic MSC with a target of 1–2 × 10^6^ cells/kg of body weight. These MSCs were specifically selected to have no repeated HLA mismatches with the kidney donor for HLA-A, HLA-B, HLA-DR, and HLA-DQ, to minimize the risk of antidonor immune response against the kidney graft. For the complete characteristics and study design, we refer to previous papers regarding the Neptune study (8, 26). In the current study, we define a couple as a recipient with their corresponding kidney and MSC donor.

### cfDNA measurements

**Figure 1:**
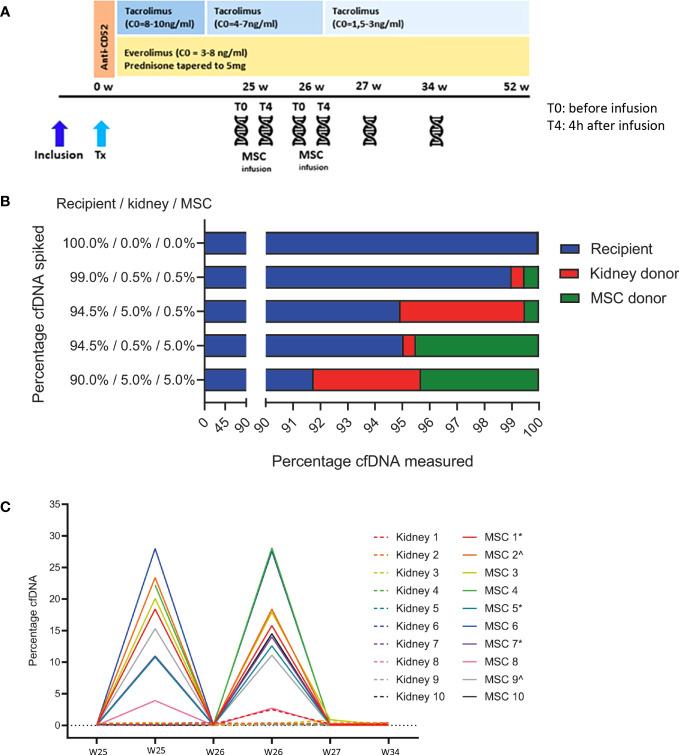
**(A)** Study timeline. The timeline of the Neptune study is shown in combination with the immunosuppressive treatment. The patient received induction therapy with alemtuzumab (Campath^®^) 15 mg on days 0 and 1 after transplantation (Tx). Maintenance therapy was everolimus (Certican^®^), tacrolimus (Advagraf^®^), and prednisone. In weeks 25 and 26, 1–2 × 10^6^/kg allogeneic MSC was infused. Cell-free DNA was measured before and 4 h after the MSC infusions in weeks 25 and 26, 27, and 34. W, weeks; Tx, transplantation; MSC, mesenchymal stromal cells; C0, trough level; 
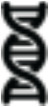
 cell-free DNA measurement. **(B)** Proportions of spiked DNA can be reproducibly detected. The genomic DNA of the recipient, kidney donor, and MSC donor was spiked in various proportions and could be reproducibly quantified by the cfDNA assay. The mean percentages of duplicate measurements are shown. MSC, mesenchymal stromal cell. **(C)** MSC-derived cfDNA levels are reproducible and elevated shortly after infusion. This figure shows the percentages of kidney donor- and MSC donor-derived cfDNA in all 10 recipients at all six time points before and after MSC infusion. Time point 1, week 25 before MSC infusion (w25 T0) from donor/recipient couple 4 is missing due to a technical failure, and time point 2, week 25 4 h after MSC infusion (w25 T4) from recipient 10 is missing due to insufficient sample volume. ^*^same MSC product used; ^^^same MSC product used. MSC, mesenchymal stromal cell.

From each patient, we selected biobanked plasma samples from six time points, except for recipient 10, for whom time point 2 was missing. The first time point was preinfusion of the MSC at week 25 after transplantation. Time point 2 was 4 h after the first infusion, and time points 3 and 4 were just before and 4 h after the second infusion, respectively (week 26). The final time points 5 and 6 were 1 and 8 weeks after the second infusion (**Figure 1A**). Plasma samples had an average volume of 1,624 µL (range: 1,100–1,800 µL). cfDNA was isolated from ethylenediaminetetraacetic acid (EDTA) plasma using a protocol developed and kindly provided by CareDx Inc. (Brisbane, CA, USA). MagMax Cell-Free DNA Isolation (Thermo Fisher, Waltham, MA, USA) was used, followed by an Ampure XP Size Exclusion Purification Procedure (Beckman Coulter, Woerden, the Netherlands). The concentration of the isolated cfDNA was determined by HS Qubit (Thermo Fisher) and diluted to 0.625 µg/µL. The average yield from the isolated cfDNA was 36.4 ng (3.5–130.9 ng).

Single-nucleotide polymorphism (SNP) typing to identify the three genomes was performed on genomic DNA. cfDNA was measured using the AlloSeq cfDNA assay (CareDx Inc.), which is a multiplexed polymerase chain reaction (PCR)-based method to prepare libraries for sequencing from cfDNA extracted from plasma. Following cfDNA extraction, the cfDNA was amplified using PCR primer pairs covering 202 SNP loci. Amplification was performed on a SimpliAmp (Thermo Fisher), followed by a short library preparation, consisting of a double bead purification and two ethanol washes. The resulting PCR products were sequenced on an Illumina MiniSeq instrument (Illumina, Eindhoven, the Netherlands) with the high-output flow cell in batches of a maximum of 24 samples. The sequencing data were analyzed using CareDx AlloSeq cfDNA software, where the read counts of amplified sequences were used to measure the amount of donor-derived cfDNA relative to the total amount of cfDNA in the plasma sample.

With the recommended cfDNA input of 10 ng, the AlloSeq cfDNA limit of detection was reported to be 0.23%. Limit of blank and limit of quantification were reported at 0.18% and 0.23%, respectively, as outlined in the assay protocol. The accuracy of the assay was previously tested with 29 unique, well-characterized samples of known % dd-cfDNA (or equivalent) and showed agreement with orthogonal results with an *R* ^2^ of 0.91362 for clinical samples, 0.945799 for analytical samples near LoD/LoQ, and 0.999138 in linearity analysis (tested up to 70%) (27, 28).

## Results

### Validation of detecting three genomes

To validate the ability to accurately determine the relative levels of three sources of cfDNA, we first prepared several genomic DNA mixtures from donor/recipient couple 7 and the corresponding MSC product that was infused. Different percentages of genomic DNA derived from donor, recipient, and MSC products were tested, with donor and MSC product cfDNA as low as 0.5%. As shown in **Figure 1B**, the test performance was very accurate. Samples were run twice with comparable results (data not shown). This validation test shows that, with known amounts of genomic DNA used, the measured values accurately represent the DNA input with a maximum deviation of 0.7%.

### MSC-derived cfDNA levels rapidly and transiently rise upon infusion

Next, we analyzed the plasma samples from all patients included in the Neptune trial.

In **Figure 1C**, the relative amount of cfDNA of the MSC product and of the kidney donor at the aforementioned six time points before and after MSC infusions is shown. MSC-derived cfDNA was detectable in all recipients 4 h after both infusions (median 18.4% and 15.2%, respectively) and was no longer detectable 1 week thereafter. MSC-derived cfDNA percentages ranged from 2.70% (recipient 8, week 26, 4 h after MSC infusion (T4)) to a maximum of 27.96% (recipient 6, week 25 T4). The level of MSC-derived cfDNA was highly consistent between the two 4-h postinfusion measurements within a patient. Importantly, kidney donor-derived cfDNA was generally within baseline levels, except for time point 1 from donor four (2.41%). This plasma sample at week 25 before infusion (T0) from donor/recipient couple 4 was contaminated with DNA from the last sample from couple number 3 at week 34 due to a technical failure. We therefore excluded this sample from the analysis. The sample at week 25 after MSC infusion from donor/recipient couple 10 is lacking due to insufficient input material.

The weight of the recipients ranged from 56.3 kg (recipient No. 5) to 93.3 kg (recipient No. 7). The mean of the number of infused MSC was 1.5 × 10^6^ MSC/kg. The highest absolute number of MSC (1.87 × 10^6^/kg = 151.47 × 10^6^ in total) was infused in recipient 10 during the second infusion, at week 26. The lowest number, 98.8 × 10^6^ in total, was infused in patient No. 1 during the first infusion at week 25 (8). The percentage of cfDNA measured did not correlate with the absolute numbers of infused MSC. Upon the highest % MSC-derived cfDNA measured in recipient 6 at week 25, a total of 119.85 × 10^6^ MSC were infused. At the lowest % MSC-derived cfDNA in recipient 8 at week 26, 121.88 × 10^6^ MSC were infused.

## Discussion

Plasma cfDNA has been investigated not only as a biomarker and readout for cell damage in several clinical settings, for example, in oncology trials, but also as a biomarker for acute rejection after solid organ transplantation (21, 29, 30). In this study, we measured cfDNA derived from three genomes in the unique situation of allogeneic MSC infused after kidney transplantation to measure MSC cell death *in vivo*. We first investigated the capacity and accuracy of the applied technique to separate three different genomes by cfDNA measurement. Until now, studies have described the separation of two different genomes, for example, derived from recipients and kidney allografts (31). With the use of allogeneic, third-party MSC, the detection of three genomes became relevant. The assay was capable of distinguishing the three genomes with sufficient accuracy.

We showed that 4 h after infusion of the MSC, there was a strong peak of MSC-derived cfDNA, consisting of up to 27.96% of all circulating cfDNA. The rapid rise in cfDNA 4 h after infusion reflects cell death of the MSC, which is no longer detectable 7 days after infusion. To our knowledge, this is the first study to investigate the cell death of MSC *in vivo* using samples from a clinical study. Our results are in line with the results from several preclinical studies, which show that MSCs are undetectable shortly after infusion (10–12). The results obtained were comparable between transplant recipients, with variability in the relative levels of MSC donor-derived cfDNA. Because relative levels of cfDNA were determined, the absolute quantity of recipient-derived cfDNA affects the contribution of donor-derived as well as MSC donor-derived cfDNA to the total cfDNA content. Indeed, in recipient 8, low but detectable levels of MSC donor-derived cfDNA were measured 4 h after infusions (3.9% and 2.70%, **Figure 1C**). Due to the characteristics of the test, a possible explanation could be a high absolute amount and therefore high percentage of recipient-derived cfDNA. Indeed, when we take into consideration the concentration of cfDNA pretransplantation (only recipient-derived), the median concentration of cfDNA was 20.52 ng/mL. Recipient number 8, with the lowest percentages of MSC-derived cfDNA, showed a pretransplant cfDNA concentration of 79.92 ng/mL. In addition, in all measurements performed for all patients, the median cfDNA level was 23.76 ng/mL, whereas only recipient number 8 had a higher median of 106.79 ng/mL cfDNA in all samples measured.

We hypothesize that the rapid and transient rise in MSC-derived cfDNA reflects cell damage and apoptosis of the MSC. The decline of MSC donor-derived cfDNA 1 week after the first and second infusions and 8 weeks after the second infusion indicates no detectable MSC cell damage or apoptosis at this time. The vast increase of cfDNA 4 h after the two infusions (median 18.4% and 15.2% of total) and decline 1 week after infusion suggest that the MSC died within 7 days, which is in line with preclinical studies (10–12). However, this does not formally rule out the possibility that a subset of MSC may survive long-term *in vivo*.

Another important result from this study is that cfDNA derived from the kidney graft was consistently low over time, in line with the results from the DART trial (median cfDNA of 0.3% of the group without ABMR) (22). Only at time point 4 in patient 1, the kidney dd-cfDNA was 2.5%. According to the DART trial, the median cfDNA measured in recipients with ABMR was 2.9%. Clinically, there was no sign of acute rejection after MSC infusion in this patient, and the protocol kidney biopsy performed in week 52 showed no kidney damage (8). Altogether, these data show that there is no detectable direct damage of the kidney graft upon MSC infusion, and this further supports the fact that during the time of observation, no undetected acute rejection episodes occurred in all patients included in the Neptune study (8).

The study has several limitations. The amount of MSC-derived cfDNA is expressed as a percentage of total cfDNA and not as an absolute amount. This means that we were not able to investigate the relative number of MSCs that died. However, the amount of MSCs infused was comparable in all patients (1.5 × 10^6^ cells/kg of body weight), so we assume that the variation in percentage MSC-derived cfDNA is due to patient factors rather than reflecting the absolute amount of degraded MSCs. In addition, as mentioned before, there is a possibility that not all MSCs have died and a subset survives. However, 7 days after infusion, no MSC-derived cfDNA was detectable in all patients. Future research with a combination of cfDNA measurement and cell labeling would probably help to address this point. Besides the fate of MSC, more insight into the actual working mechanism is interesting. Previously, it has been shown that apoptosis, dependent on IDO activity, seems to be an important factor for the immunomodulatory effect of MSC (10). To gain more insight, it would be interesting to compare IDO activity with MSC-derived cfDNA values. Unfortunately, in the current study, we were not able to measure IDO activity. In addition, previous research showed that infusion of MSC could trigger an inflammatory cascade leading to complement activation and activation of immune effector cells, commonly summarized as an instant blood-mediated inflammatory reaction (IBMIR) (32–34). This process of IBMIR could lead in a more active way to the destruction of the MSC and early degradation of MSC after infusion. Combining the monitoring of IBMIR after infusion with the measurement of cfDNA could give more insight into the mechanism of degradation of MSC after infusion.

Since this is the first study to separate three genomes with cfDNA measurement, this sets the stage for further research in transplantation, especially in studies using third-party products. Furthermore, this study shows that in the setting of clinical kidney transplantation, MSCs perish shortly after infusion, suggesting that their mechanism of action is an indirect process. It is important to further investigate this process, as it may open the possibility of developing therapies specifically directed at the downstream processes. This may ultimately result in the development of cell-free treatments with the same results but without the risks associated with cellular therapies.

## Data availability statement

The datasets presented in this article are not readily available due to storage restrictions of the original data from the Neptune study, following the rules of the CCMO. Requests to access the datasets should be directed to the principal investigator of the study.

## Ethics statement

The studies involving humans were approved by the local ethics committee and by the Central Committee on Research involving Human Subjects. The studies were conducted in accordance with the local legislation and institutional requirements. The participants provided their written informed consent to participate in this study.

## Author contributions

GD: analyzed the data and wrote the manuscript. JD: performed the experiments. JF, CK, MR, and SH: designed the study and co-wrote the manuscript. All authors contributed to the article and approved the submitted version.
